# Laparoscopic Repair of Traumatic Diaphragmatic Hernia Secondary to Gunshot Wound

**DOI:** 10.7759/cureus.77548

**Published:** 2025-01-16

**Authors:** Sofia Aramburu, Camila Bras Harriott, Francisco Schlottmann

**Affiliations:** 1 General Surgery, Hospital Aleman, Buenos Aires, ARG

**Keywords:** gunshot wound, laparoscopic hernia repair, minimally invasive surgery, trauma surgery, traumatic diaphragmatic hernia

## Abstract

This report presents the case of a 40-year-old man with a history of emergency surgery due to a gunshot wound. Twelve years later, he presented with persistent symptoms, including epigastric pain, pyrosis, and dysphagia. Diagnostic studies reveal a complex paraesophageal and paracardial diaphragmatic hernia. Traumatic diaphragmatic hernias (TDHs) present diagnostic challenges, particularly during the latent phase, with symptoms ranging from gastrointestinal to respiratory. A comprehensive evaluation and correct diagnosis are required, including diagnostic modalities such as CT scans, upper endoscopy, and esophagogastroduodenal transit. Surgical approaches, whether laparoscopic or thoracoscopic, are chosen based on individual preference for adhesions. The report highlights the success of laparoscopic management, emphasizing early detection to prevent complications during both latent and obstructive phases of TDH. Additionally, it emphasizes the necessity of regular follow-ups for trauma patients.

## Introduction

Traumatic diaphragmatic hernia (TDH) is a rare yet significant complication of thoracic or abdominal trauma. It occurs when a defect in the diaphragm allows abdominal organs to herniate into the thoracic cavity. TDH may result from either blunt or penetrating trauma, such as motor vehicle accidents, stab wounds, or gunshot wounds (GSWs), with penetrating injuries accounting for a significant proportion of cases. Despite its potentially life-threatening nature, TDH can remain undiagnosed for years, particularly in the latent phase when symptoms are absent or nonspecific.

The clinical presentation of TDH varies widely. Patients may experience gastrointestinal symptoms, such as nausea, vomiting, dysphagia, or epigastric pain, and respiratory symptoms, such as dyspnea or chest pain. In some cases, the condition may only be detected incidentally during imaging studies conducted for unrelated reasons. Advanced imaging techniques, such as computed tomography (CT), and diagnostic tools, such as barium swallow studies and esophagogastroduodenoscopy (EGD), play a crucial role in confirming the diagnosis by delineating the herniated structures and identifying diaphragmatic defects [1]. However, diagnostic laparoscopy is the most effective diagnostic tool in cases of strong clinical suspicion, as small diaphragmatic hernias can be missed on cross-sectional imaging. Moreover, in high diagnostic suspicion and hemodynamic decompensation, exploratory laparotomy should not be delayed.

Management of TDH is predominantly surgical and aims to reduce herniated contents and repair the diaphragmatic defect with tension-free repair and selective use of prosthetic mesh, especially in cases of large defects. The surgical approach should be individualized according to the patient's presentation and surgeon experience. Laparoscopic approach is feasible if the patient is stable, whereas open approach is useful where concomitant injuries are present or if the contents of hernia are gangrenous [2]. Minimally invasive approaches, such as laparoscopic or thoracoscopic techniques, are a good treatment option in experienced hands for chronic TDH due to their lower morbidity and faster recovery times [3]. According to the American Association for the Surgery of Trauma (AAST), the approach to repairing a diaphragmatic hernia varies based on the size of the defect. Smaller defects can typically be repaired using simple sutures, while larger or more complex defects often require the use of prosthetic materials, such as mesh, to ensure a secure and durable repair. The choice of repair technique may also depend on additional factors, such as the patient's overall condition, the presence of associated complications, and the availability of advanced surgical techniques such as laparoscopy or thoracoscopy [4].

## Case presentation

**Figure 1:**
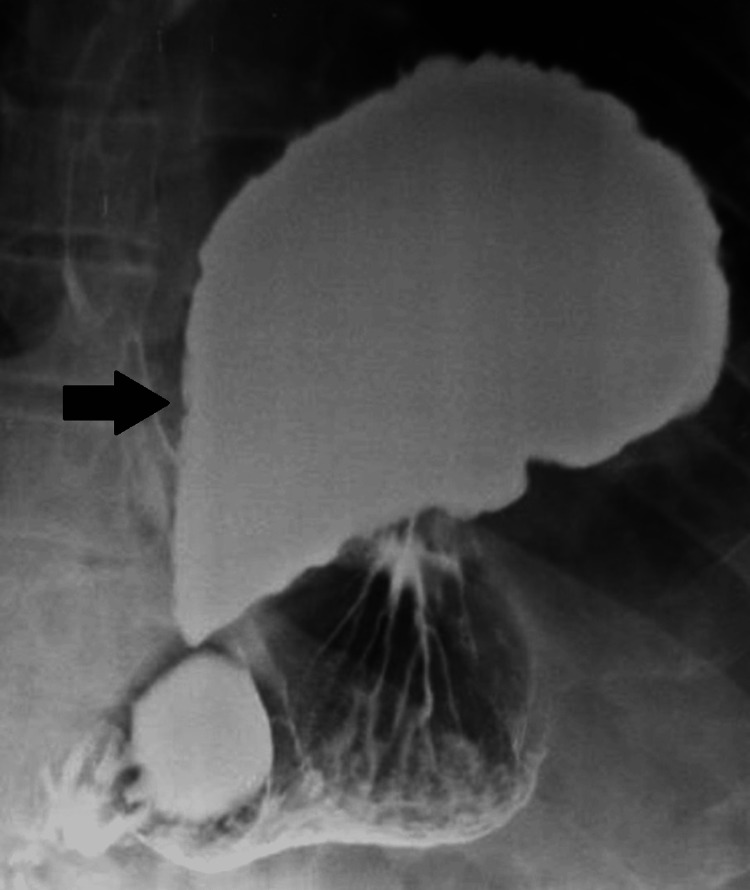
Barium swallow Voluminous left parahiatal hernia. The entire gastric fundus is herniated in the thoracic cavity (black arrow).

**Figure 2:**
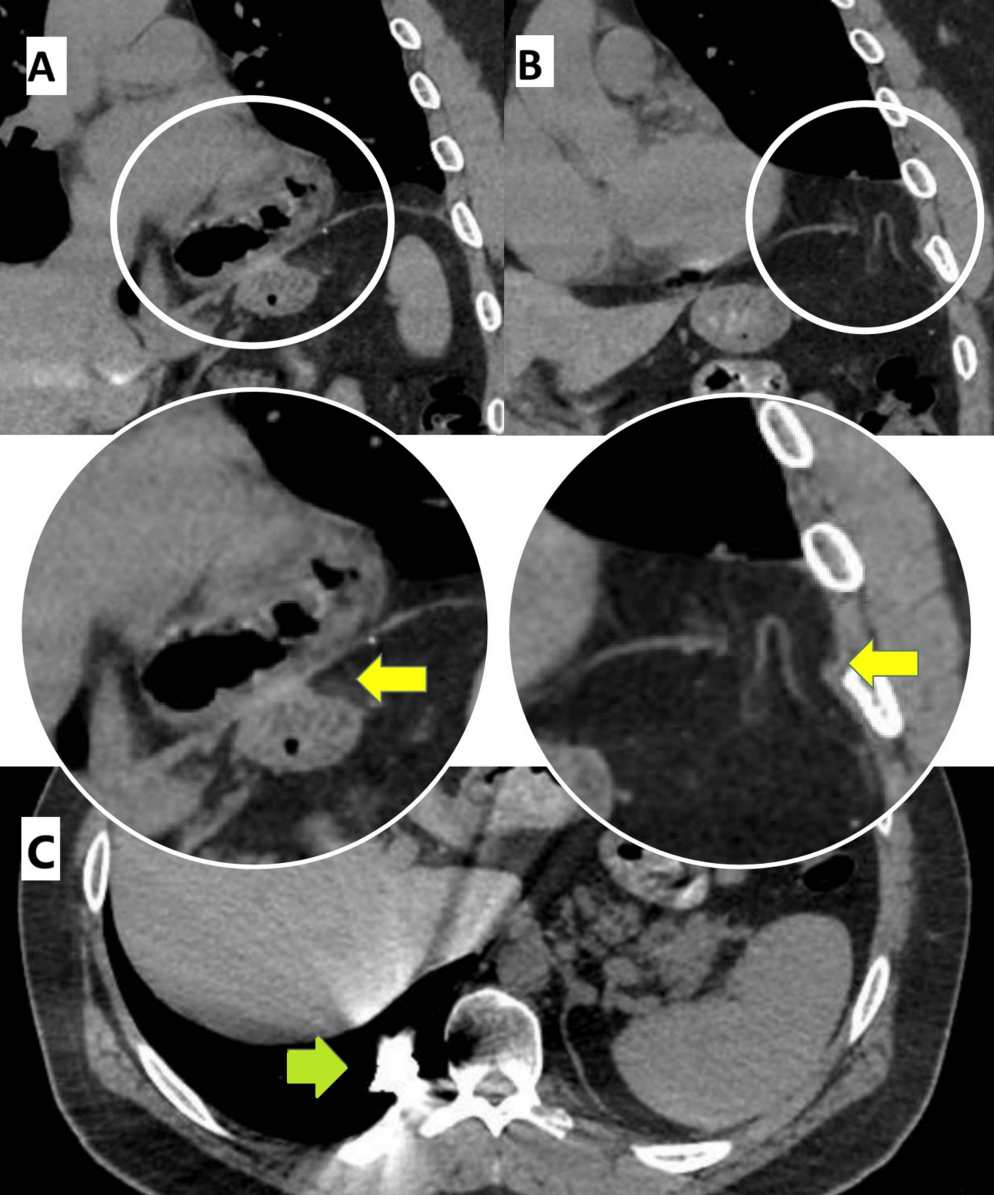
Thoracic and abdominal CT scan A. Voluminous left parahiatal diaphragmatic hernia with gastric fundus in the thoracic cavity (yellow arrow). B. Left subcostal diaphragmatic hernia with adipose content (yellow arrow). C. Metal density image in the posterobasal segment of the right lower lobe (green arrow).

A 40-year-old man with a history of prior emergent thoracotomy and laparotomy after GSW 12 years ago at another institution presented with complaints of intermittent epigastric pain associated with heartburn, dysphagia, and regurgitation for the last six months. We decided to perform a barium swallow, esophagogastroduodenoscopy (EGD), and thoracic and abdominal computed tomography (CT). The barium swallow showed a large diaphragmatic hernia with the entire gastric fundus located in the left thoracic cavity (Figure 1). The EGD revealed a parahiatal diaphragmatic hernia with sliding of the gastric fundus, associated with erosive gastropathy and blood remnants corresponding to esophageal Cameron ulcers. The CT showed a large left parahiatal diaphragmatic hernia with sliding of the gastric fundus (Figure 2A) and a smaller diaphragmatic defect with adipose content just above the spleen (Figure 2B). A metal density image in the posterobasal segment of the right lower lobe corresponding with the history of previous GSW was also identified (Figure 2C).

**Figure 3:**
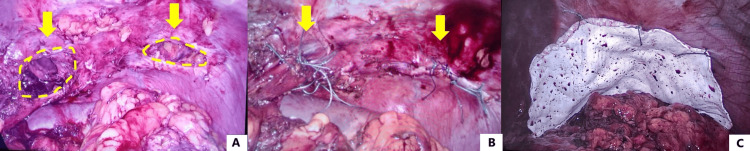
Laparoscopic repair of traumatic diaphragmatic hernia A. Identification of both diaphragmatic defects: left parahiatal and left subcostal (yellow arrow and circles). B. Diaphragmatic defects closed with interrupted non-absorbable sutures (yellow arrows). C. Reinforcement of the repair with a polytetrafluoroethylene mesh covering both defects.

Based on these findings, a laparoscopic repair of diaphragmatic hernia was planned. A balanced general anesthesia was performed, and an 18 g venous access was placed. Induction was done with propofol, using remifentanil as an analgesic and rocuronium as a muscle relaxant. Maintenance of anesthesia was done with sevoflurane, remifentanil, and rocuronium depending on the need for relaxation or neurostimulation. Selective intubation was performed to facilitate comfortable surgical manipulation and to avoid lung injury during the procedure. Multimodal analgesia was performed, consisting of opioids (morphine) and NSAIDs (ketorolac and paracetamol). During the exploratory laparoscopy, both diaphragmatic defects were identified: left parahiatal with the gastric fundus and left subcostal with greater omentum (Figure 3A). The greater omentum was reduced into the abdominal cavity, identifying a diaphragmatic subcostal defect of approximately 4 cm in diameter. The esophageal hiatus was then dissected, recognizing the right and left crus of the diaphragm and the esophagus. The esophagus was encircled with a Penrose drain to avoid its injury and to facilitate dissection of the parahiatal defect. The gastric fundus was gently reduced with blunt maneuvers and an ultrasonic scalpel, identifying a parahiatal defect of approximately 5 cm. Both defects were closed with non-absorbable interrupted sutures (i.e., Polyester 2-0) (Figure 3B). A 15x20 cm reinforcing mesh of polytetrafluoroethylene (a synthetic fluoropolymer of tetrafluoroethylene that has numerous applications because it is chemically inert) was then placed covering both defects, which was fixed to the diaphragm with non-absorbable interrupted sutures (Figure 3C). The postoperative course was uneventful, and the patient was discharged on postoperative day 2 tolerating soft diet adequately. After a follow-up of six months, the patient remains asymptomatic.

## Discussion

TDH is the direct consequence of either thoracic or abdominal trauma. The creation of different gradients of pressure or direct diaphragm rupture usually leads to the passage of abdominal structures into the thoracic cavity. The principal herniated organs in order of frequency are the stomach, colon, omentum, spleen, small bowel, and left kidney in left-sided hernias, and the liver, colon, small bowel, and omentum in right-sided hernias. TDH can be classified into three categories: acute, from the moment of the trauma until the recovery of the patient; latent, after the recovery from initial injuries (the patient may be symptomatic or not); and obstructive, after full recovery the patient presents acute obstruction symptoms such as incarceration, ischemia, necrosis or perforation [1]. Blunt trauma after motor vehicle accidents and penetrating trauma are the main causes of TDH [5]. Penetrating trauma is usually related to stab wounds or GSWs and represents 61%-73% of cases. In cases of acute trauma, surgical exploration with laparoscopy or thoracoscopy (if found in specialized centers with trained surgeons) or with an open approach should be one of the first diagnostic studies to be performed, especially in acutely unstable patients or those with highly suspicious symptoms [3,6].

Prior history of trauma along with typical gastrointestinal symptoms (nausea, vomiting, early satiety, epigastric pain, dysphagia, regurgitation) or respiratory symptoms (dyspnea, chest pain) can help reach the diagnosis. Some patients, however, are asymptomatic, and the TDH is incidentally diagnosed [7]. The CT is the most useful imaging study because it allows for the proper identification of the diaphragmatic defect and identification of the structures herniated to the thoracic cavity. A barium swallow can also help better delineate the anatomy of the herniated stomach. EGD is useful in identifying potential mucosal lesions in the herniated stomach.

Surgical repair is recommended in symptomatic patients. The approach can be either laparoscopic or thoracoscopic, depending mostly on the surgeon's preference. In some cases, a hybrid thoracoscopic and laparoscopic approach might be needed [8]. In our case, laparoscopic exploration was preferred, and there was no need to use the thoracoscopic approach intraoperatively. Minimally invasive surgery is often the operative modality of choice for most abdominal and pelvic surgery, especially due to advantages such as reduced morbidity and faster recovery times [9]. The main objective of the operation is to reduce all the herniated structures into the abdominal cavity and close the defect without tension. Reinforcement of the repair with a prosthetic mesh is recommended in patients with large defects where performing a primary closure generates a tension closure. In addition to that, our patient had two hernial defects, approximately 4 and 5 centimeters each, with a separation of 6 centimeters between each defect; therefore, we considered the use of a mesh necessary [10,11]. The peri-operative insertion of a chest tube depends on the open communication between the surgeon and anesthesiologist.

## Conclusions

The main purpose of this case report is to highlight the importance of recognizing and managing TDHs, a condition that can remain latent for several years following trauma, posing significant diagnostic and therapeutic challenges. The successful laparoscopic repair of diaphragmatic defects in this patient demonstrates the effectiveness of minimally invasive approaches combined with the use of prosthetic mesh to ensure durable outcomes. A correct diagnosis, facilitated by imaging and endoscopic studies, is critical to prevent complications associated with delayed presentations, although diagnostic confirmation is performed with surgical exploration, especially in acute presentations with unstable patients or stable patients with high diagnostic suspicion. Diagnostic laparoscopy offers a diagnostic and therapeutic tool to prevent progression of occult traumatic diaphragmatic injuries to chronic diaphragmatic hernias.
